# CircRNA: the potential biomarkers and therapeutic targets in oral squamous cell carcinoma (OSCC)

**DOI:** 10.3389/fonc.2025.1555002

**Published:** 2025-06-05

**Authors:** Jun-Lin Lv, Ru Ma, Yu-Shan Ren, Qing-Yue Liang, He-Meng Zhang, Gui-Cheng Dong, Jie Li

**Affiliations:** ^1^ College of Pharmacy, Inner Mongolia Medical University, Hohhot, Inner Mongolia Autonomous Region, China; ^2^ School of Basic Medicine, Qingdao University, Qingdao, Shandong, China; ^3^ School of Stomatology, Zhangzhou Health Vocational College, Zhangzhou, Fujian, China; ^4^ College of Life Sciences, Inner Mongolia Agricultural University, Hohhot, Inner Mongolia Autonomous Region, China

**Keywords:** circular RNA, oral squamous cell carcinoma, tumorigenesis, mechanism, biomarkers

## Abstract

Circular RNAs (circRNAs) are a new category of non-coding RNAs implicated in the molecular pathology of cancer, such as oral squamous cell carcinoma (OSCC). Circular intronic RNAs (ciRNAs), exonic circRNAs (ecircRNAs), and exon-intron circRNAs (EIciRNAs) are three primary types of circRNAs resulted from the circularization of extron and intron which give rise to the distinct biology of circRNAs. Due to their unique structure and biogenesis, circRNAs exhibit tissue- and cell-specific expression profiles. Recent studies have highlighted that in OSCC, some circRNAs are differentially expressed compared with adjacent normal tissues, with these variables potentially influencing OSCC initiation and progression through diverse mechanisms. Furthermore, earlier clinical trials have indicated that circRNAs could be considered as potential therapeutic targets and biomarkers for OSCC. It should be noted that many circRNAs modulate tumor cells proliferation/apoptosis and metastasis via regulating gene transcription and post transcriptional expressions. Furthermore, certain circRNAs function as effective microRNA sponges, thereby inhibiting oncogenic pathways in OSCC. In summary, the discovery of circRNAs has unveiled new avenues for cancer research, particularly in OSCC. This review provides an overview of circRNA biogenesis, their biological functions, and their roles as differentially expressed molecules in OSCC, emphasizing their potential for clinical application and warranting further investigation into their functional and therapeutic relevance.

## 1 Introduction

Oral squamous cell carcinoma (OSCC) is a malignant tumor that is prevalent in the head and neck region, ranking eleventh among the most common cancers worldwide (1). OSCC arises from various risk factors, including smoking, chronic betel nut chewing, human papillomavirus (HPV) infection, and poor oral hygiene, typically affecting areas such as the tongue, gums, oropharynx, and buccal mucosa (2, 3). Due to its asymptomatic and non-specific early-stage presentation, OSCC is often diagnosed at advanced stages, frequently with lymph node metastasis (LNM) or distant metastasis (DM) (4). Conventional surgical approaches often fail to achieve complete remission, necessitating adjunctive therapies like radiotherapy or chemotherapy to improve survival and prognosis (5). However, the inherent tumor heterogeneity allows cancer cells to frequently develop resistance to these treatments, with approximately 60% of patients with OSCC showing resistance to radiation or chemotherapy in clinical settings (6). Given these complexities, the current clinical strategies for OSCC remain suboptimal, with a five-year survival rate below 60% (7). This underscores the urgent need to elucidate the molecular mechanisms driving OSCC in order to identify effective biomarkers and novel therapeutic targets for clinical application.

The discovery of new disease targets and diagnostic technologies has brought the prospect of tumor eradication closer to reality due to recent advances in biology (8). For instance, identify the non-coding RNAs (ncRNAs), verify their specific biological functions has challenged the previous notion of these RNAs as mere ‘genetic byproducts’ (9). Circular RNAs (circRNAs) are endogenous non-coding RNAs that are covalently closed loop structures and are expressed everywhere and specifically in eukaryotic cells. Initially considered byproducts of aberrant splicing (10), circRNAs are now recognized for their stability, as their unique closed-loop structure protects them from degradation by nucleases (11). Furthermore, the circRNAs that are expressed differently in different diseases and tumor tissues indicate their tissue- and time-specific functions (10, 12). In addition, exosomes secreted by oral cancer stem cells also contain circRNAs, which may similarly influence OSCC progression through intercellular communication (13). Although many aspects of circRNA biology remain unclear, accumulating evidence suggests that certain circRNAs may serve as promising therapeutic targets and diagnostic/prognostic biomarkers in cancers. This review provides an overview of the biological activities and mechanisms of circRNAs, highlighting their vital roles in the tumorigenesis of OSCC, examining their regulatory mechanisms, and exploring their potential clinical applications.

## 2 CircRNAs synthesis (biogenesis)

**Figure 1:**
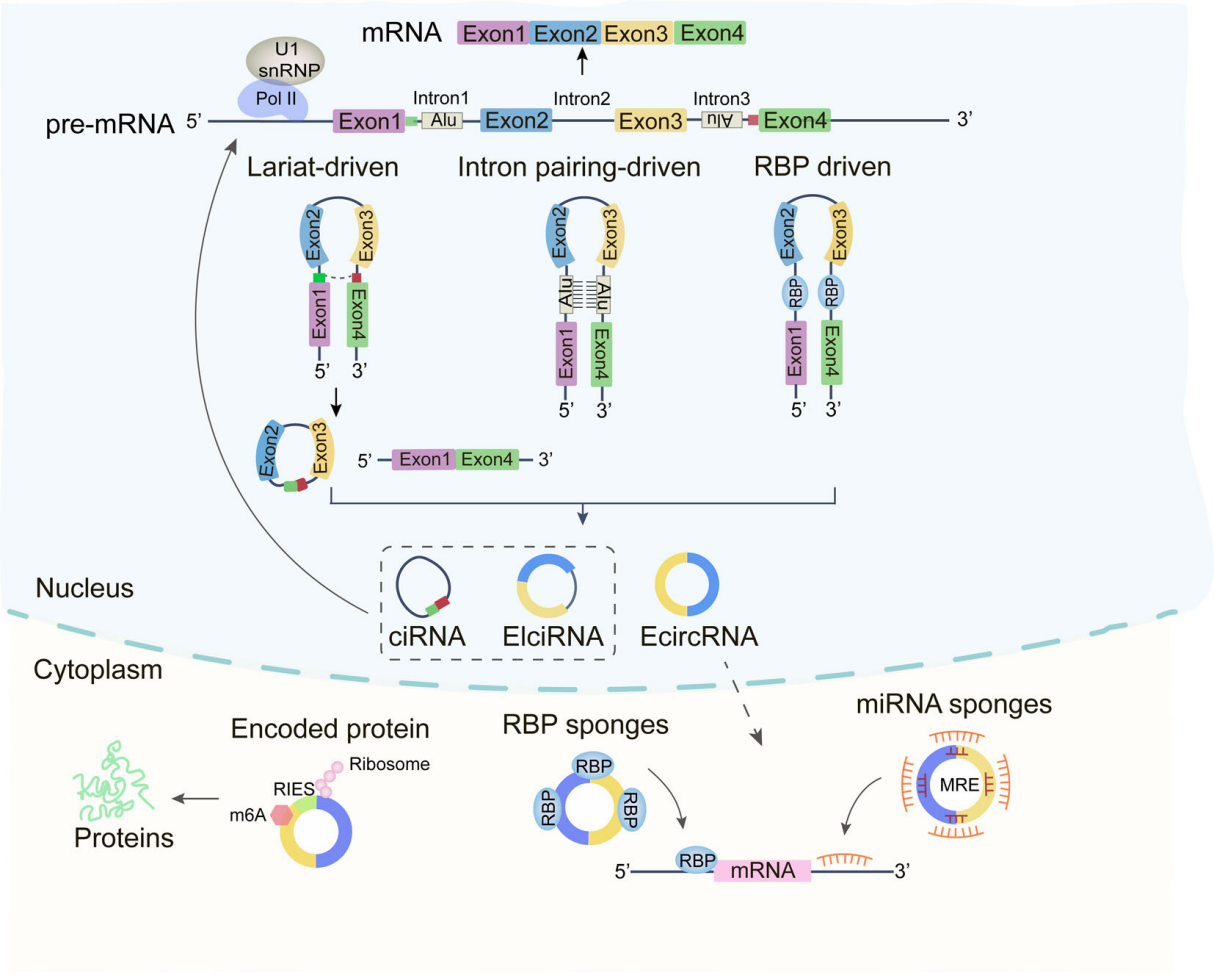
Depiction of the biogenesis and functions of circRNAs. Circular RNAs (circRNA) are synthesized via back-splicing, culminating in a covalently bonded, closed cyclic nucleotide structure. These circRNAs play a pivotal role in interplaying with RNA Binding Proteins (RBP), acting as sponges for miRNAs, and vying with mRNAs in the modulation of gene expression and protein translation.

In eukaryotes, newly synthesized precursor mRNAs consist of introns and exons that undergo splicing to produce various RNA forms (14). In classical RNA splicing, the upstream 5’-donor site is linked to the downstream 3’-acceptor site, where introns are removed and exons are ligated to form mature linear mRNAs (15). In contrast, circRNA formation involves reverse splicing, where the 3’ end of an exon joins the 5’ end of an intron, creating a covalently closed circular structure composed of one or more exons, devoid of a 5’-cap or 3’-poly(A) tail (16, 17). After splicing, circRNAs can be formed three main types based on their sequence features: exon-derived circRNAs (ecRNAs), intronic circRNAs (ciRNAs), and exon-intron complex circRNAs (EIciRNAs). Most circRNAs originate from exons and are principally distributed in the cytoplasm, while circRNAs containing intronic sequences are more abundant in the nucleus (18, 19). Their formation mechanisms can be categorized as follows (**Figure 1**).

### 2.1 Circularization driven by exon skipping

Exon skipping leads to the generation of both linear mRNA and a lasso-like structure comprising skipped exons and introns (17). Reverse splicing of this lasso can produce three types of circRNAs: EIciRNA, ecRNA, and ciRNA (20).

### 2.2 Intron base pairing

Some circRNAs arise from matching complementary sequences by base-pairing in the flanking intronic regions of exons, followed by alternative splicing to form EIciRNAs or ecRNAs (21).

### 2.3 RBP-mediated circularization

RNA-binding proteins (RBPs) binding to sites within the flanking intronic regions of exons can bring the splice donor and acceptor sites into proximity, facilitating circRNA circularization (22).

## 3 Functions of circRNAs

The subcellular localization of circRNAs determines their functional diversity (23). Most circRNAs reside in the cytoplasm, where they function as miRNA sponges, regulating target mRNA expression through competitive binding to miRNAs at specific 3’UTR regions (24). Some cytoplasmic circRNAs also recruit ribosomes for translation into proteins or peptides (16). In contrast, nuclear circRNAs are involved in regulating gene transcription (18).

### 3.1 miRNA sponges

Cytoplasmic circRNAs, which contain multiple miRNA response elements (MREs), competitively sequester miRNAs, preventing them from binding to mRNA 3’UTRs and thus modulating mRNA stability (25). For instance, CircHIPK3, widely expressed in human cells, sponges multiple miRNAs (e.g., miR-30a-3p, miR-7, and miR-124-3p), influencing processes such as tumor growth, metastasis, and angiogenesis (26–28).

### 3.2 Interaction with RBPs

RBPs are integral to the regulation of gene expression. Certain circRNAs, characterized by RBP-binding motifs, can form stable loops through complementary sequences, interacting with proteins to exert their functions (29, 30). For example, the RBP human antigen R (HuR), a key player in colorectal cancer, associates with circRHOBTB3, promoting its ubiquitination and degradation, which in turn reduces PTBP1 mRNA levels and suppresses tumor metastasis (31). In LCC and LLN cells, circMTCL1 interacts with the RBP protein C1Q binding protein (C1QBP), inhibiting its ubiquitination and degradation, therefore regulating the tumor progression through Wnt/β-catenin signaling pathway (32).

### 3.3 Translation (coding)

The cap-dependent ribosome scanning mechanism is the primary mode of translation in eukaryotes (33). While circRNAs were once thought to be non-translatable but recent findings imply that some circRNAs possess internal ribosome entry sites (IRES) or m6A modifications in the 5’-UTR, enabling translation (34). In breast cancer, circSEMA4B both encodes the protein SEMA4B-211aa and sponge miR-330-3p, inhibiting the phosphorylation of the PI3K/AKT pathway in tumors (35). In stomach cancer, circMAPK1 encodes a peptide of 109 amino acids, which binds to and inhibits MEK1 function (36).

### 3.4 Regulation of parental gene expressions

Nuclear-localized EIciRNAs and ciRNAs interact with Pol II (RNA polymerase II) or U1 snRNP (small nuclear proteins) and regulating parental gene expression (22). For instance, circSMARCA5 is downregulated in breast cancer while its parental gene SMARCA is upregulated. circSMARCA5 binds to its parental gene to form an R-loop, inhibiting transcription and regulating DNA damage repair and cisplatin resistance (37). Similarly, CircME1 binds to U1 snRNP at the promoter of its parental gene *ME1*, positively regulating its expression and promoting renal cell carcinoma progression (38).

## 4 CircRNAs in OSCC

**Figure 2:**
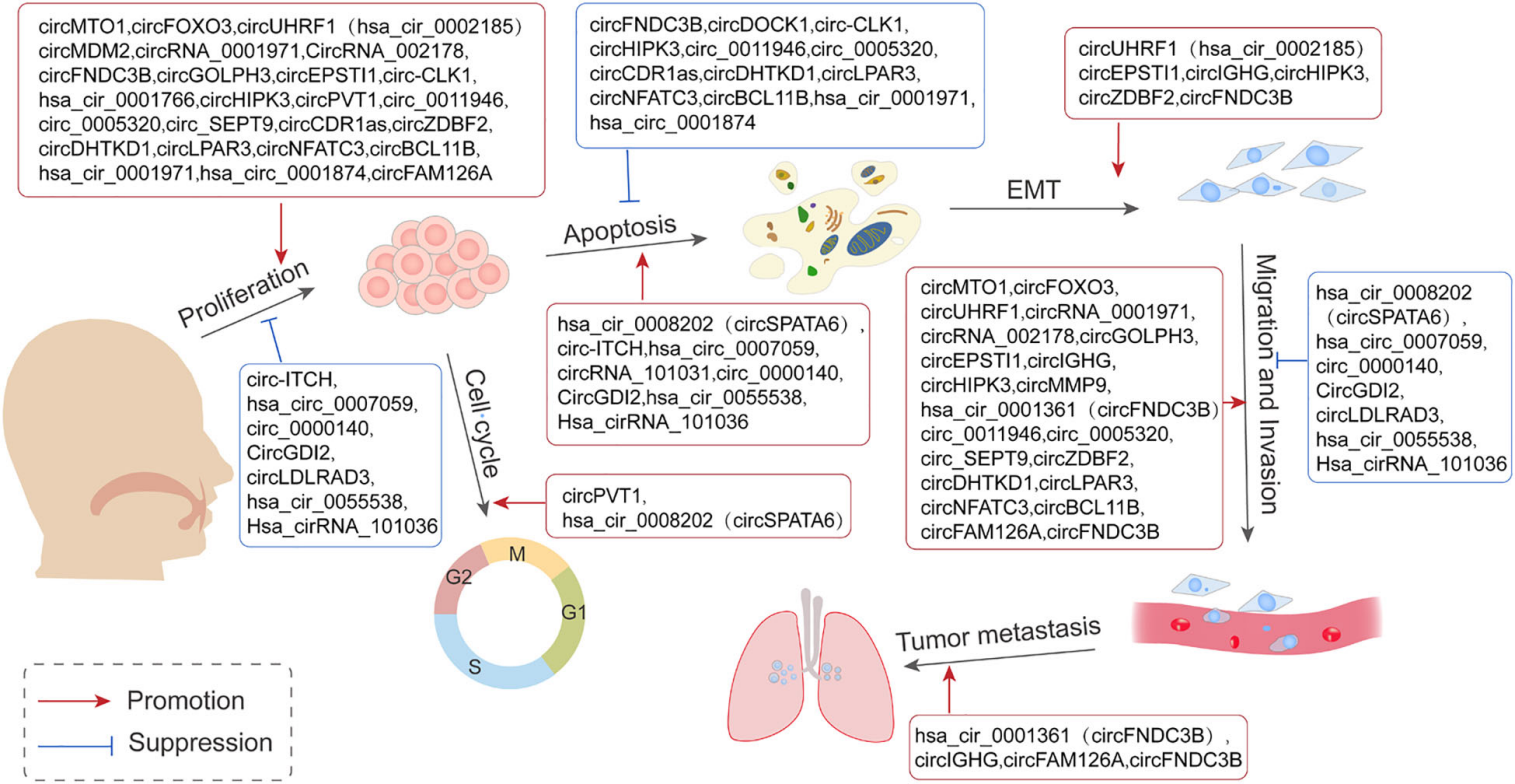
Representation of the contribution of circRNAs in the mediation of various stages of Oral Squamous Cell Carcinoma (OSCC) tumorigenesis. This is achieved primarily through the mechanism of acting as sponges for miRNAs and in turn regulating the expression of pertinent genes involved in cell proliferation, apoptosis, cycle arrest, invasion, and metastasis.

Commonly used methods for detecting circRNAs in tissues include microarray analysis and RNA sequencing. In addition, single-cell sequencing technology enables the detection of circRNAs at the single-cell level, making it particularly suitable for uncovering cellular heterogeneity. Studies on OSCC have identified 43 differentially expressed circRNAs (30 upregulated and 13 downregulated), elucidating their roles in the tumorigenesis and progression of OSCC (**Table 1**). Mechanistically, many circRNAs act as miRNA sponges, modulating gene expression across various stages of OSCC development, including cell proliferation, apoptosis, cycle arrest, invasion, metastasis, angiogenesis, immune response, and drug resistance (**Figure 2**).

**Table 1:** The expression and characteristic features of circRNAs in OSCC.

circBase_ID (name, expression*)	Spliced source (length)	Targets	Anti-tumor effects				Cell lines	Ref.
			A	I	M	P		
hsa_circ_0007874 (circMTO1, **↑**)	Exons 5-6 (304)	miR-320a/ATRX	✓	✓	✓	✓	CAL-27 HSC-3	(39)
hsa_circ_0006404 (circFOXO1, ↑)	Exons 5 (1,435)	miR-214/KDM2A		✓		✓	SCC-4 SCC-9	(40)
hsa_circ_0002185 (circUHRF1, ↑)	Exons 12-13 (301)	miR-526b-5p/c-Myc	✓	✓	✓	✓	SCC-25 CAL-27	(41)
hsa_circ_0027451 (circMDM2, ↑)	Exons 4-7 (669)	miR-532-3p/HK2				✓	SCC-25 CAL-27	(42)
			Glycolysis (+)					
hsa_circ_0001971 (circFAM126A, ↑)	Exons 7-12 (575)	miR-186-5p/FNDC3B miR-186/SHP2		✓	✓	✓	SCC-4 HSC-3 SCC-9	(43) (44)
hsa_circ_0001682 (circFAM126A, ↑)	Exons 12-13 (181)	miR-186-5/RAB41		✓	✓	✓	CAL-27 UM1	(45)
hsa_circ_0000519 (circRPPH1, ↑)	Exons 1 (98)	Akt/mTOR		✓	✓	✓	SCC-25 CAL-27	(46)
hsa_circ_0001361 (circFNDC3B, ↑)	Exons 2–3 (215)	miR-520d-5p/SLC7A11 MDM2/FUS/HIF1A miR-181c-5p/PROX1		✓	✓	✓	CAL-27 SCC-15 HSC-3	(47) (48)
			Ferroptosis (-)					
			Angiopoiesis (+)					
hsa_circ_0001470 (circGOLPH3, ↑)	Exons 2–3 (247)	miR-1299/LIF		✓	✓	✓	UM1 HN4	(49)
hsa_circ_0000479 (circEPSTI1, ↑)	Exons 9-13 (375)	miR-942-5p/LTBP2		✓		✓	CAL-27 SCC-9	(50)
hsa_circ_0020396 (circDOCK1, ↑)	Exons 3-27 (2,514)	miR-196a-5p/BIRC3	✓				CAL-27 SCC-9	(51)
hsa_circ_0000579 (circIGHG, ↑)	Exons 2-9 (27,944)	miR-142-5p/IGF2BP3		✓	✓	✓	CAL-27	(52)
hsa_circ_0577725 (circCLK1, ↑)	Exons 7-11 (509)	miR-18b-5p/YBX2				✓	UM1 HSC-2	(53)
hsa_circ_0001766 (circPDIA4, ↑)	Exons 8-9 (387)	miR-877-3p/VEGFA				✓	SCC-9 SCC-25	(54)
hsa_circ_0000284 (circHIPK3, ↑)	Exons 4 (1,099)	miR-637/NUPR1/PI3K		✓	✓	✓	Tca-8113 SCC-9	(55)
hsa_circ_0001821 (circPVT1, ↑)	Exons 3 (410)	miR-125b/STAT3				✓	CAL-27 SCC-9	(56)
hsa_circ_0011946 (circSCMH1, ↑)	Exons 11-16 (782)	miR-216a-5p/BCL2L2		✓	✓	✓	CAL-27 SCC-25	(57)
			Cisplatin sensitivity (+)					
hsa_circ_0005320 (circSEPT9, ↑)	Exons 10-11 (645)	miR-486-3p/JAK2 miR-1225/PKN2		✓	✓	✓	CAL-27 SCC-25 UM1 SCC-15	(58) (59)
hsa_circ_0001946 (circCDR1as, ↑)	Exons 1 (1,485)	miR-671-5p/mTOR				✓	Tca-8113 SCC-15	(60)
			Autophagy (+)					
hsa_circ_0002141 (circZDBF2 ↑)	Exons 2-5 (290)	miR-362-5p/RNF145		✓	✓	✓	SCC-9 SCC-15	(61)
hsa_circ_0007813 (circDHTKD1, ↑)	Exons 2-10 (2,504)	miR-326/GAB1		✓	✓	✓	SCC-9 Cal-27	(62)
hsa_circ_0004390 (circLPAR3, ↑)	Exons 3-4 (754)	miR-144-3p/LPCAT1		✓	✓	✓	SCC-25 HSC-3	(63)
			Angiogenesis (+)					
			Glycolysis (+)					
hsa_circ_0005615 (circNFATC3, ↑)	Exons 2 (1,135)	miR-520h/LDHA		✓	✓	✓	SCC-25 HSC-3	(64)
			Glycolysis (+)					
hsa_circ_0033144 (circBCL11B, ↑)	Exons 4 (369)	miR-579/LASP1		✓	✓	✓	CAL-27 SCC-9	(65)
hsa_circ_0001874 (circBICD2, ↑)	Introns 1 (304)	miR-296-5/PLK1				✓	SCC-9	(44)
hsa_circ_0001162 (circMMP9, ↑)	Exons 12-13 Introns 12 (328)	miR-149/AUF1		✓	✓	✓	UM1 HSC-3	(66)
hsa_circ_0007294 (circANKS1B, ↑)	Exons 5-8 (459)	miR-515-5p/TGF-β1		✓	✓	✓	CAL27 SCC9 SCC090	(67)
			Cisplatin sensitivity (+)					
hsa_circ_0060927 (circCYP24A1, ↑)	Exons 4-13 (1,106)	miR-195-5p/TRIM14		✓	✓	✓	SCC-9 SCC-25	(68)
hsa_circ_0000199 (circAKT3, ↑)	Exons 8-11 (555)	miR-145-5p miR-29b-3p				✓	SCC-9 HN12	(69)
hsa_circ_0069313 (circPACRGL, ↑)	Exons 1-8 Introns 1-7 (2,073)	miR-325-3p/PDL1	–				CAL-27SCC-9	(70)
			Immune escape (+)					
hsa_circ_0008202 (circSPATA6, ↓)	Exons 5-7 (285)	miR-182/TRAF6		✓	✓	✓	CAL-27 HSC-6	(71)
hsa_circ_0001141 (circITCH, ↓)	Exons 7-14 (873)	miR-421/PDCD4	✓				SCC-6 HN4	(72)
hsa_circ_0007059 (circZNF720, ↓)	Exons 5-6 (223)	AKT/mTOR	✓	✓	✓	✓	SCC-15 CAL-27	(73)
hsa_circ_0000140 (circKIAA0907, ↓)	Exons 7-10 (585)	miR-31/LATS2		✓	✓	✓	Cal-27 HSC-3	(74)
			Radiosensitivity (+)					
hsa_circ_0005379 (circGDI2, ↓)	Exons 2-5 (674)	miR-454-3p/FOXF2 miR-424-5p/SCAI	✓	✓	✓	✓	SCC-15 HSC-3 CAL-27	(75) (76)
			Glycolysis (-)					
			Cetuximab sensitivity (+)					
hsa_circ_0006988 (circLDLRAD3, ↓)	Exons 5 (346)	miR-558/SMAD4		✓	✓	✓	SCC-9 SCC-15	(77)
hsa_circ_0025765 (circTMTC1, ↓)	Exons 17-18 (1,006)	ER	✓	✓	✓		OECM-1 HSC-3	(78)
hsa_circ_0070401 (circPKD2, ↓)	Exons 4-7 Introns 3-7 (1,189)	miR-646/ATG13	✓				SCC-15 CAL-27	(79)
			Autophagy (-)					
			Cisplatin sensitivity (+)					
hsa_circ_0020093 (circATRNL1, ↓)	Exons 4-7 (1,137)	miR-23a-3p/PTEN	✓			✓	HSC3 HSC6 CAL27	(80)
			Radiosensitivity (+)					
hsa_circ_0006877 (circLDLR, ↓)	Exons 13-14 (295)	miR-1178	✓	✓	✓		HSC3 SA3	(4)
hsa_circ_009755 (circVWA8, ↓)	Exons 13-16 (–)	–	–				–	(81)
hsa_circ_0093229 (circTRDMT1, ↓)	Exons 3-8 (713)	–	–				–	(79)
hsa_circ_0086414 (circBNC2, ↓)	Exons 6 (1,970)	–	–				–	(82)

*Tumor vs. Normal; A, Apoptosis; I, Invasion; M, Migration; P, Proliferation; ↑ means: Compared with normal tissues, circRNA was upregulated in OSCC; ↓ means: Compared with normal tissues, circRNA was downregulated in OSCC.

## 5 CircRNAs promote the proliferation, invasion, and migration in OSCC

Metastasis represents the final stage of cancer progression, including in OSCC (5). Factors such as the tumor microenvironment, genetic predispositions, and mutations contribute to the transformation of normal epithelial cells into tumor cells with excessive proliferative capacities. These transformed cells form clonal subtypes and acquire the ability to invade, spread, and colonize distant organs (83). Excessive proliferation thus serves as a critical driver of tumor development. Lymphatic and distal metastases resulting from unchecked proliferation are major contributors to the high recurrence and mortality rates associated with OSCC (84). Previous studies have pointed out the anti-tumorigenesis effects of circRNA-miRNA axis in OSCC through regulating the downstream target gene expressions (85). Key signaling pathways influenced by this axis are as follows.

### 5.1 Transforming growth factor beta-SMADs signal pathway

**Figure 3:**
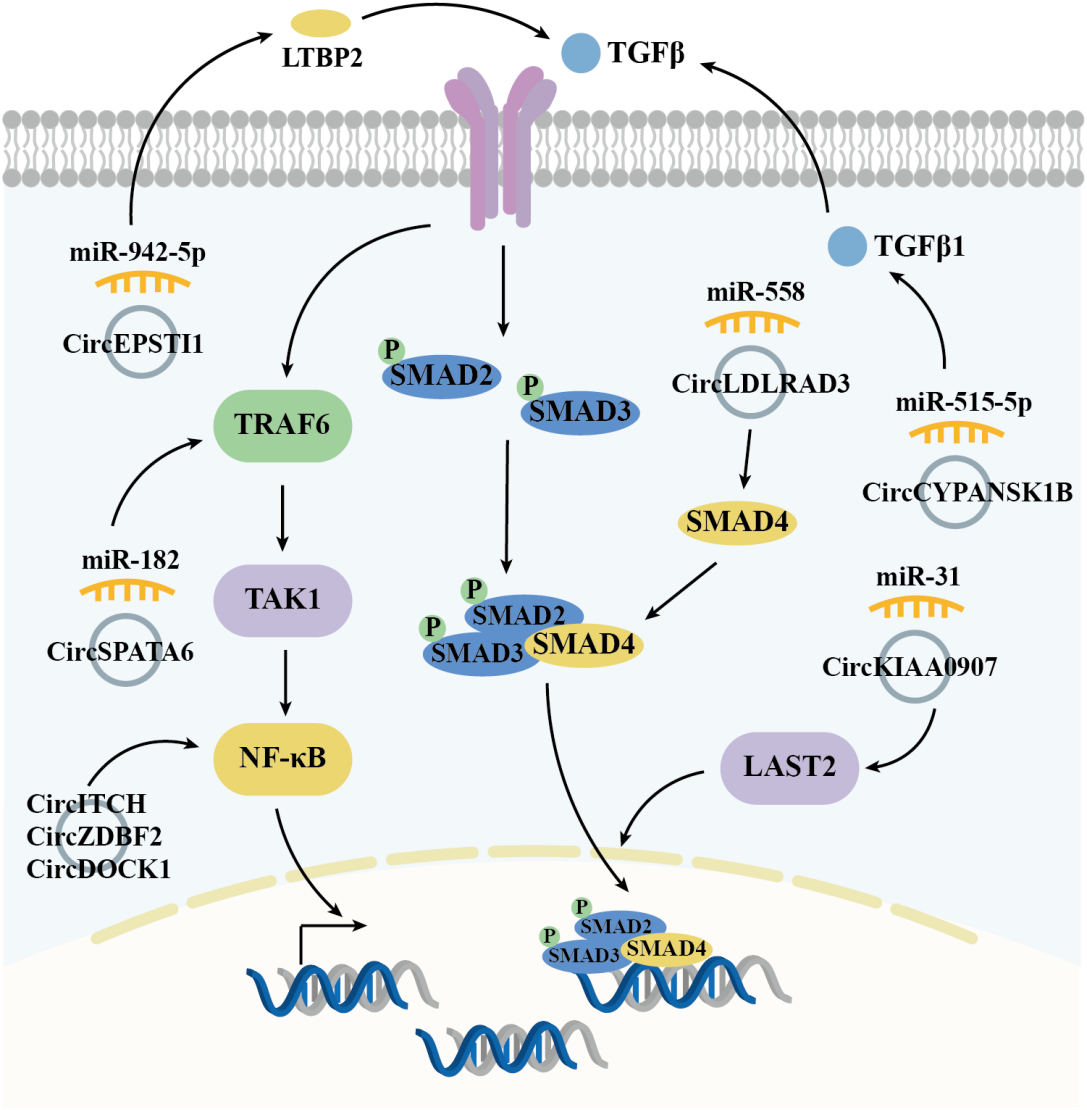
Diagrammatic illustration of how circRNAs regulate the TGFβ-SMADs signaling pathway in the mediation of OSCC.

The TGFβ signaling pathway, which includes extracellular cytokines, cytomembrane receptors, and intracellular signal messengers, is essential for regulating cell behavior. TGFβ receptors (TGFβ R) recruit and activate downstream transcription factors, SMAD family member 2/3 (SMAD2/3), which, in conjunction with SMAD4, translocate to the nucleus. There, they regulate gene transcription, influencing the cell cycle distributions, cell proliferation/apoptosis, adhesion, and metastasis. In OSCC, overexpressed circRNAs, such as circEPSTI1 (50) and circCYPANSK1B (67), promote LTBP2 (Latent Transforming Growth Factor Beta Binding Protein 2) and TGFβ1 expressions by sponging miR-942-5p and miR-515-5p, respectively. This activation of the TGFβ pathway facilitates OSCC cell proliferation, metastasis, and invasion. Conversely, circKIAA0907 (74) and circLDLRAD3 (77) act as potential suppressors of OSCC. Their low expression in OSCC tissues leads to a failure to sponge miR-31 and miR-558, resulting in the indirect inhibition of LATS2 (Large Tumor Suppressor Kinase 2) and SMAD4. TAK1 (TGF-β activated kinase 1), a pivotal regulatory factor in cell death by activating downstream effectors, including NF-κB (Nuclear Factor Kappa B) and MAPKs (mitogen-activated protein kinases). Over-expressed circSPATA6/miR-182 axis has been shown to activate TAK1/NF-κB pathway by upregulating TRAF6 (TNF Receptor Associated Factor 6) expression, thereby promoting the tumorigenesis in OSCC cells (71). As a key transcription factor, NF-κB expression and/or activity is also indirectly regulated by circRNAs in OSCC, including through the circDOCK1/miR-196a-5p/BIRC3 (51), circZDBF2/miR-362-5p/RNF145 (61), and circITCH/miR-421/PDCD4 (72) axes, all of which contribute to OSCC tumorigenesis (**Figure 3**).

### 5.2 Phosphatidylinositol-3-kinase-AKT signal pathway

**Figure 4:**
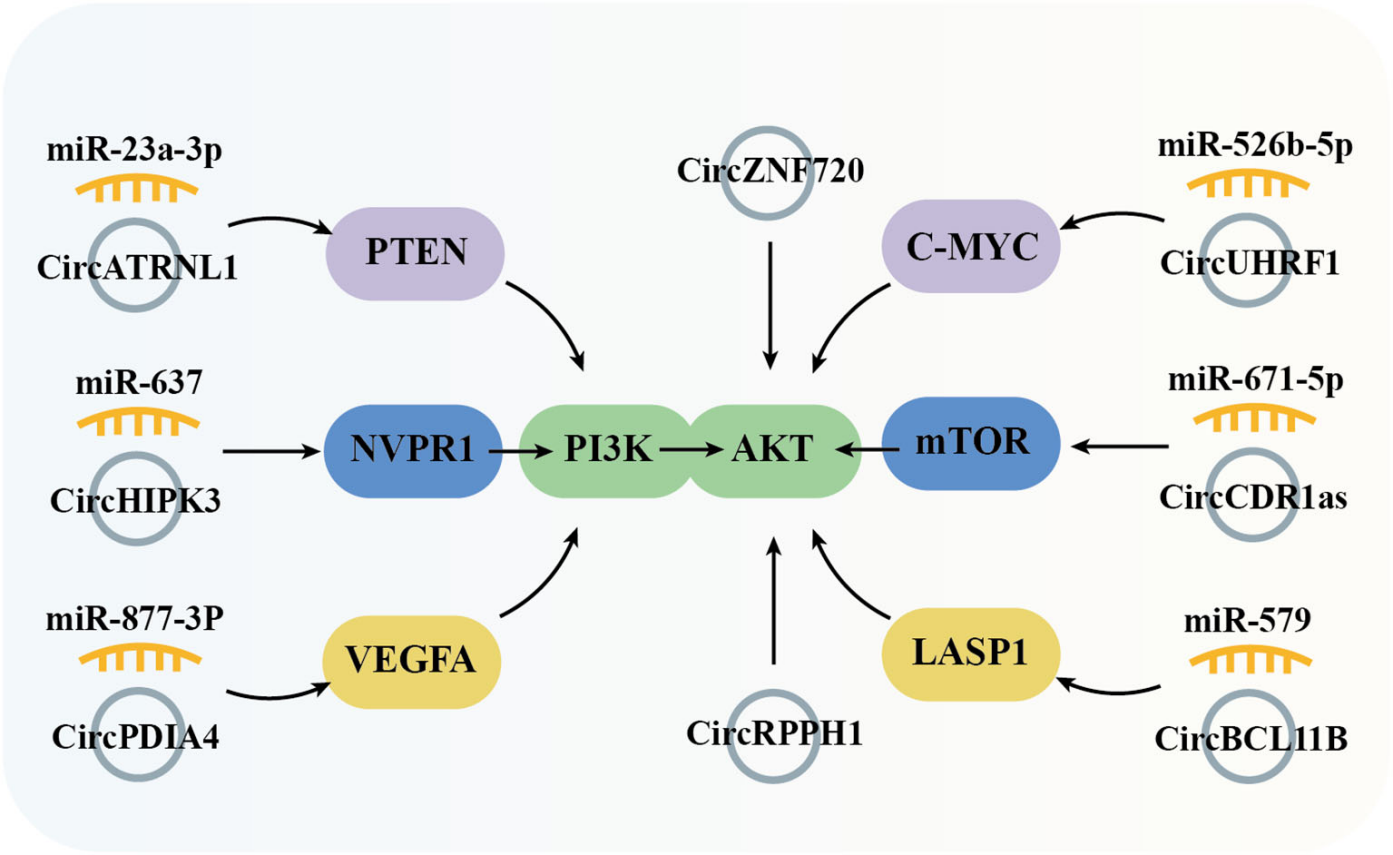
Graphical representation of circRNA-mediated OSCC through the regulation of the PI3K/Akt signaling pathway.

The PI3K/Akt signaling pathway, activated by RTKs (receptor tyrosine kinases), is essential in controlling the onset and progression of numerous cancers, including OSCC, by altering metabolism, cell proliferation, survival, and angiogenesis (**Figure 4**). Studies have demonstrated that the circPDIA4/miR-877-3p axis, which is overexpressed in OSCC, promotes VEGFA (vascular endothelial growth factor A) expression. This, in turn, activates PI3K through its receptor, driving the proliferation of OSCC cell lines SCC9 and SCC25 (54). Similarly, circHIPK3, which is highly expressed in OSCC, sponges miR-637 to induce the expression of NUPR1 (nuclear protein 1), thereby activating PI3K and promoting OSCC cell proliferation, metastasis, and invasion (55). PTEN (phosphatase and tensin homolog) is a PI3K pathway negative regulatory factor by inhibiting PI3K activity. In OSCC tissues, the expression of circATRNL1 is significantly lower compared to adjacent tissues, which impairs its ability to sponge miR-23a-3p. This results in reduced PTEN expression, leading to increased PI3K activation and enhanced tumor progression (80). In OSCC, PI3K activation leads to phosphorylation of AKT (protein kinase B, PKB), facilitating downstream signaling. CircRPPH1 (46) and circZNF720 (73) directly upregulate AKT expression, promoting cell proliferation and metastasis in OSCC tissues and cell lines. LASP1 (LIM and SH3 protein 1), a gene associated with lymph node metastasis and poor clinical prognosis, is upregulated in several malignant tumors, underscoring its oncological significance. In OSCC, circBCL11B/miR-579 regulates LASP1 expression, which activates AKT, thereby influencing OSCC tumorigenesis and progression (65). c-MYC, a PI3K/AKT pathway downstream transcription factor, is indirectly upregulated by the circUHRF1/miR-526b-5p axis, contributing to cell cycle distribution, proliferation, apoptosis, and cell differentiation (41). In addition to c-MYC, mTOR (mammalian target of rapamycin) is another critical kinase, is activated by the PI3K-AKT pathway. mTOR regulates metabolism, immune response, autophagy, and cell survival. In OSCC, circRPPH1 and circCDR1as, both highly expressed in tumor tissues, modulate mTOR activity by activating AKT and sponging miR-671-5p, respectively, thereby promoting OSCC cell proliferation, metastasis, and invasion (46, 60).

### 5.3 Janus kinase-signal transducer and activator of transcription signal pathway

**Figure 5:**
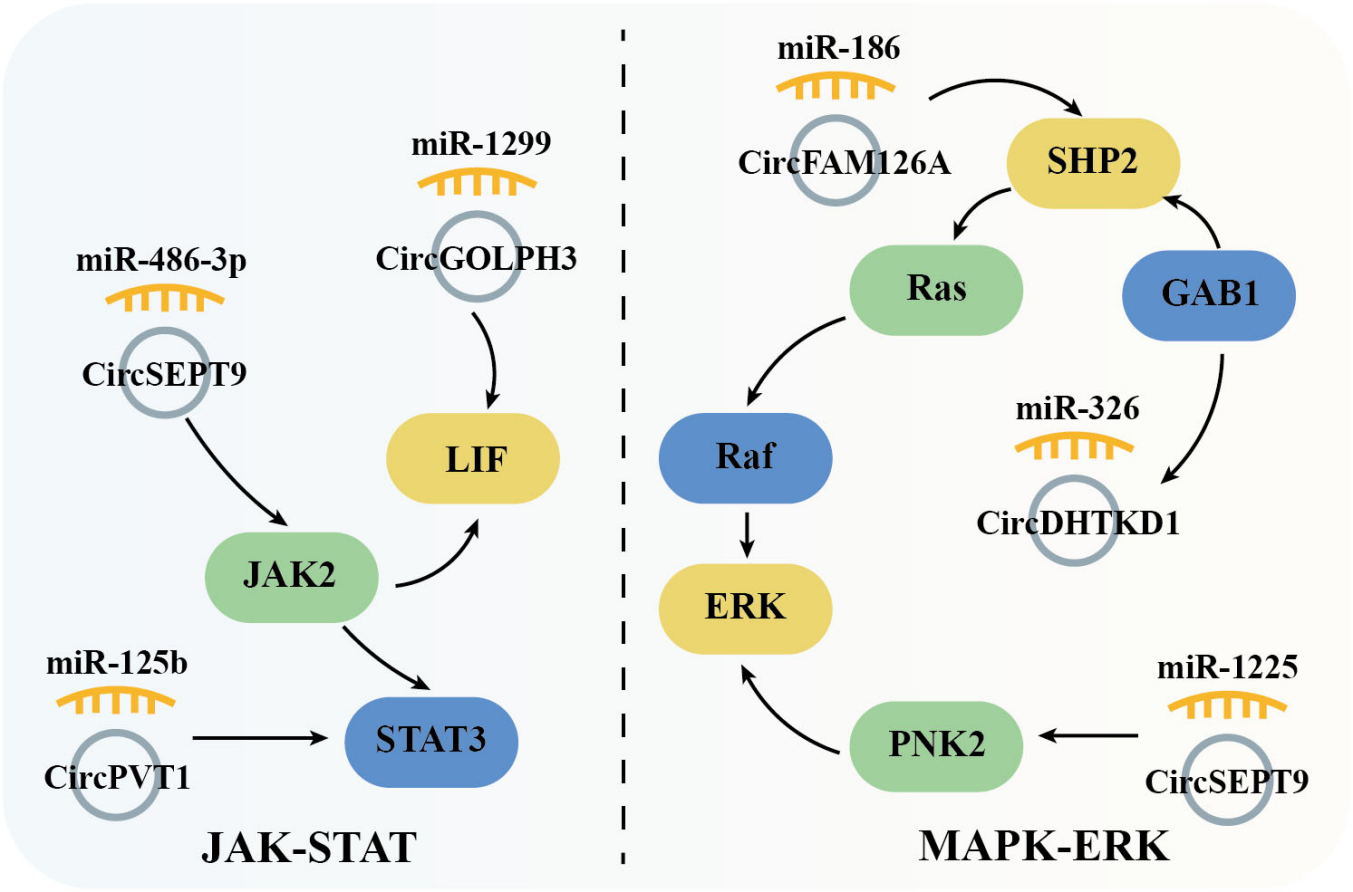
Schematic elucidation of circRNAs’ regulation of the JAK-STAT and MAPK-ERK signaling pathway and their consequential mediation of OSCC.

The JAK/STAT signaling pathway plays an important role in hematopoiesis, immunity, tissue repair, inflammation, apoptosis, and adipogenesis. This signaling pathway encompasses over 50 members, including IFNs (Interferons), ILs (Interleukins), CSFs (Colony stimulating factors), and hormones. Dysregulation or mutations in JAK/STAT components are implicated in numerous human diseases, including OSCC. Studies have shown that circSEPT9 (56) and circPVT1 (58) are highly expressed in OSCC tissues and regulate the expression of JAK2 and STAT3 through miR-486-3p and miR-125b, respectively, promoting cell proliferation and metastasis. Additionally, the circGOLPH3/miR-1299 axis induces the expression of Leukemia inhibitory factor (LIF), which activates JAK signaling and contributes to OSCC progression (49) (**Figure 5**).

### 5.4 Mitogen-activated protein kinase-ERK signal pathway

The MAPK cascade is critical for regulating normal cell proliferation, survival, and differentiation, with dysregulation often leading to cancer and other diseases. Specifically, as a crucial member of MAPK pathway, ERK (extracellular signal-regulated kinase) is activated by Raf serine/threonine kinases, which serve as downstream effectors of the commonly mutated oncogene Ras small GTPase. This forms the Ras/Raf/ERK signaling network, which is further modulated by the tyrosine phosphatase SHP2 to regulate cell proliferation, survival, and differentiation. In OSCC, overexpressed circFAM126A promotes SHP2 expression by sponging miR-186 [54]. Similarly, circDHTKD1 and circSEPT9, which are also highly expressed in OSCC, upregulate GAB1 (GRB2-associated binding protein 1) and PKN2 (protein kinase N2) through miR-326 and miR-1225, respectively. GAB1 directly activates SHP2, while PKN2 activates ERK, thus driving OSCC cell proliferation, metastasis, and invasion (59, 62) (**Figure 5**).

### 5.5 Glycolysis

Cancer cells reprogram their metabolism to support cell growth, metastasis, and survival. Increased glucose uptake and reliance on glycolysis are essential for meeting the synthetic metabolic demands of these cells (86). This tumor cell specific metabolic characteristics is defined as ‘Warburg effect,’ persists even in the presence of fully functional mitochondria (87). Hexokinase 2 (HK2), a rate-limiting enzyme in glycolysis, plays a key role in tumorigenesis. HK2 is high expressed in OSCC compared with adjacent normal tissues, and has been shown to promote OSCC cell growth both *in vitro* and *in vivo* (88). Previous investigation suggested that circMDM2 is significantly upregulated in OSCC, promote OSCC proliferation and glycolysis through regulating the miR-532-3p/HK2 axis (42). In addition, knockout of LDHA (lactate dehydrogenase A) inhibits cell proliferation and EMT process in OSCC cells (89). Similarly, circNFATC3 is over-expressed in OSCC which acts as a miR-520h sponge. This interaction induces LDHA expression and promotes glycolysis, proliferation, and invasion in OSCC cells (64). Conversely, circGDI2 is low expressed in OSCC. When overexpressed, circGDI2 targets miR-424-5p/SCAI axis, regulates glycolytic proteins like GLUT1 and LDHA, and therefore inhibiting OSCC cell reproduction and metastasis (76).

**Figure 6:**
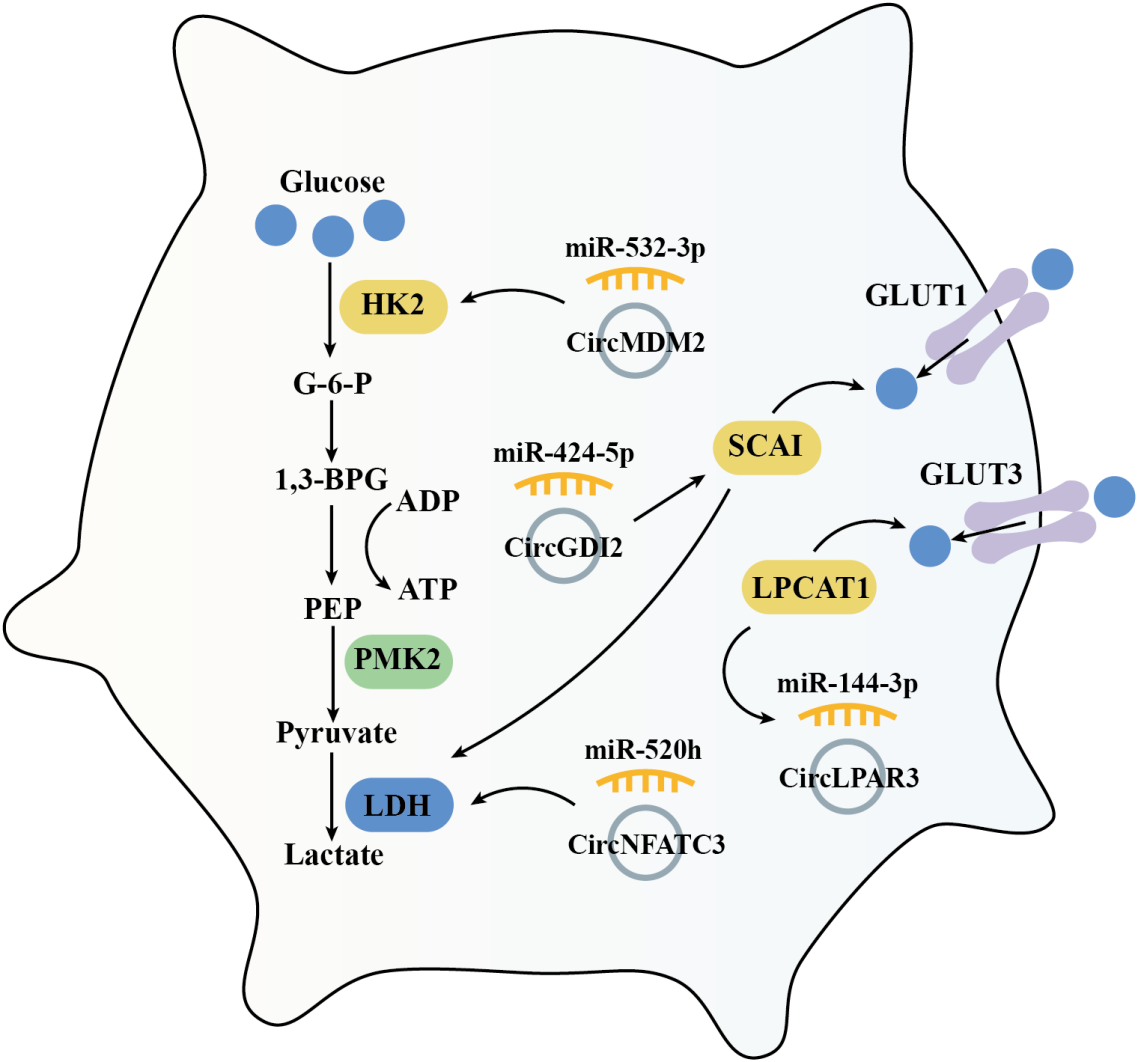
Diagram indicating how circRNAs govern the glycolysis process in the mediation of OSCC.

LPCAT1 (Lysophosphatidylcholine acyltransferase 1) is an enzyme involved in phospholipid biosynthesis and remodeling, playing a pivotal role in the lipid remodeling and various cancers, including OSCC (90, 91). In OSCC, LPCAT1 promotes tumorigenesis *via* regulating PAF (platelet-activating factor) and its receptor, PAFR (92). Furthermore, LPCAT1 has been shown to activate the NF-κB/STAT3 signal pathway and then increased GLUT3 expression, enhanced glycolysis, and increased proliferation in keratinocytes (93). In OSCC tissues and cell lines, overexpressed circLPAR3 *via* miR-144-3p promotes LPCAT1 which enhances OSCC cell proliferation, migration, invasion, angiogenesis, and glycolysis (63) (**Figure 6**).

### 5.6 Autophagy and Ferroptosis

**Figure 7:**
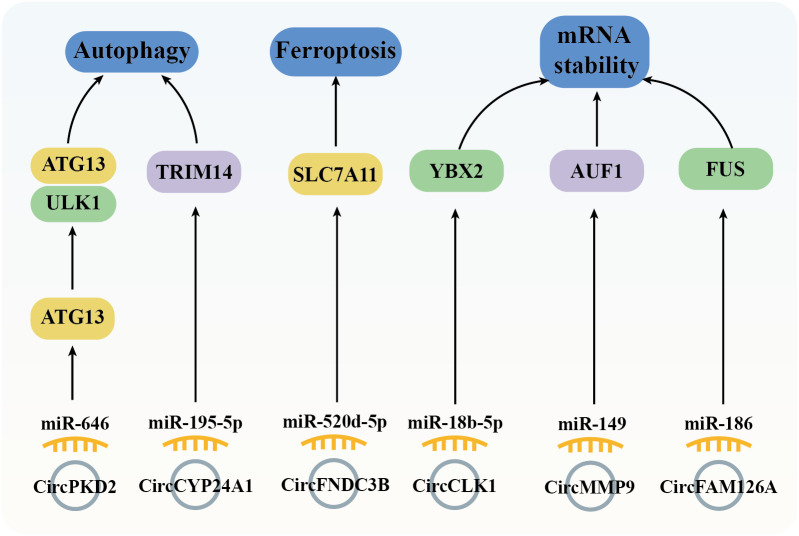
Diagram depicting the regulation of autophagy, ferroptosis, and mRNA stability by circRNAs in the mediation of OSCC.

Autophagy is a highly conserved cellular process essential for maintaining homeostasis through self-digestion and catabolism (94). In tumor cells, autophagy involved in regulating both cell survival and apoptosis signaling (95). ATG13 (autophagy-related protein 13) is a key component of the ULK1 complex, whom could activate ULK1 kinase activity (96). In tumor cells, ATG13 knockout impedes cell cycle progression and inhibits proliferation in both *in vitro* and *in vivo* models (97). Furthermore, downregulation of circPKD2 fails to sponge miR-646, leading to suppressed ATG13 expression and reduced tumorigenesis in OSCC tissues and cell lines (79). In addition to ATG13, TRIM14 (tripartite motif 14) has been implicated in mediating HCC cells proliferation, autophagy, and metastasis, with TRIM14 knockdown resulting in the opposite effects (98). In OSCC, the circCYP24A1/miR-195-5p axis upregulates TRIM14 expression, driving carcinogenesis (68) (**Figure 7**).

Ferroptosis, a regulated cell death (RCD) process, is triggered by the toxic lipid peroxidation products (99). The evasion of ferroptosis through oncogenes and oncogenic signals promotes tumor initiation, progression, metastasis, and drug resistance (100). In cancer cells, dysregulated expression of the cysteine transporter SLC7A11 (Solute Carrier Family 7 Member 11) imports cystine, biosynthesis GSH, thereby inhibiting ferroptosis (101). In OSCC tissues, high expression of circFNDC3B modulates the miR-520d-5p/SLC7A11 axis to prevent ferroptosis and promote tumor survival (48) (**Figure 7**).

### 5.7 mRNA stability

Genetic variations are widely recognized as drivers of cancer; however, post-transcriptional events also significantly influence cancer development by regulating mRNA cycling and translation, including mRNA stability, which is controlled by RBPs (102). Notable RBPs such as FUS (Fused in Sarcoma), YBX2 (Y-box Binding Protein 2), and AUF1 (AU-rich Element RNA-binding Protein 1) could regulate proliferation, migration, and invasion across various tumor cell types (102–104). Previous studies have demonstrated that overexpression of circCLK1 (53), circMMP9 (66), and circFAM126A (45), through sponging specific miRNAs (miR-18b-5p, miR-149, and miR-186-5p, respectively), enhances the expression of YBX2, AUF1, and FUS, thereby promoting OSCC tumorigenesis (**Figure 7**).

### 5.8 Other factors

In addition to regulating the tumorigenesis related signal pathways, dysregulated circRNAs in OSCC tissues and/or cell lines have also been implicated could modulate transcription factor expressions. For example, circIGHG is overexpressed in OSCC tissues and is associated with poor prognosis in OSCC patients. In terms of mechanism, circIGHG induces IGF2BP3 expression *via* binding to miR-142-5p, thereby promoting OSCC cell invasiveness (52). Furthermore, the oncogenic circFNDC3B serves multiple roles in OSCC. In the nucleus, circFNDC3B co-localizes with FUS, facilitating FUS ubiquitination and degradation. Through regulation of MDM2 and HIF1A expression, it positively influences VEGF expression and angiogenesis. In the cytoplasm, circFNDC3B regulates the miR-181c-5p/PROX1 axis to promote lymphangiogenesis and tumor metastasis (47).

## 6 CircRNAs and radiotherapy/chemotherapy resistance in OSCC cells

**Figure 8:**
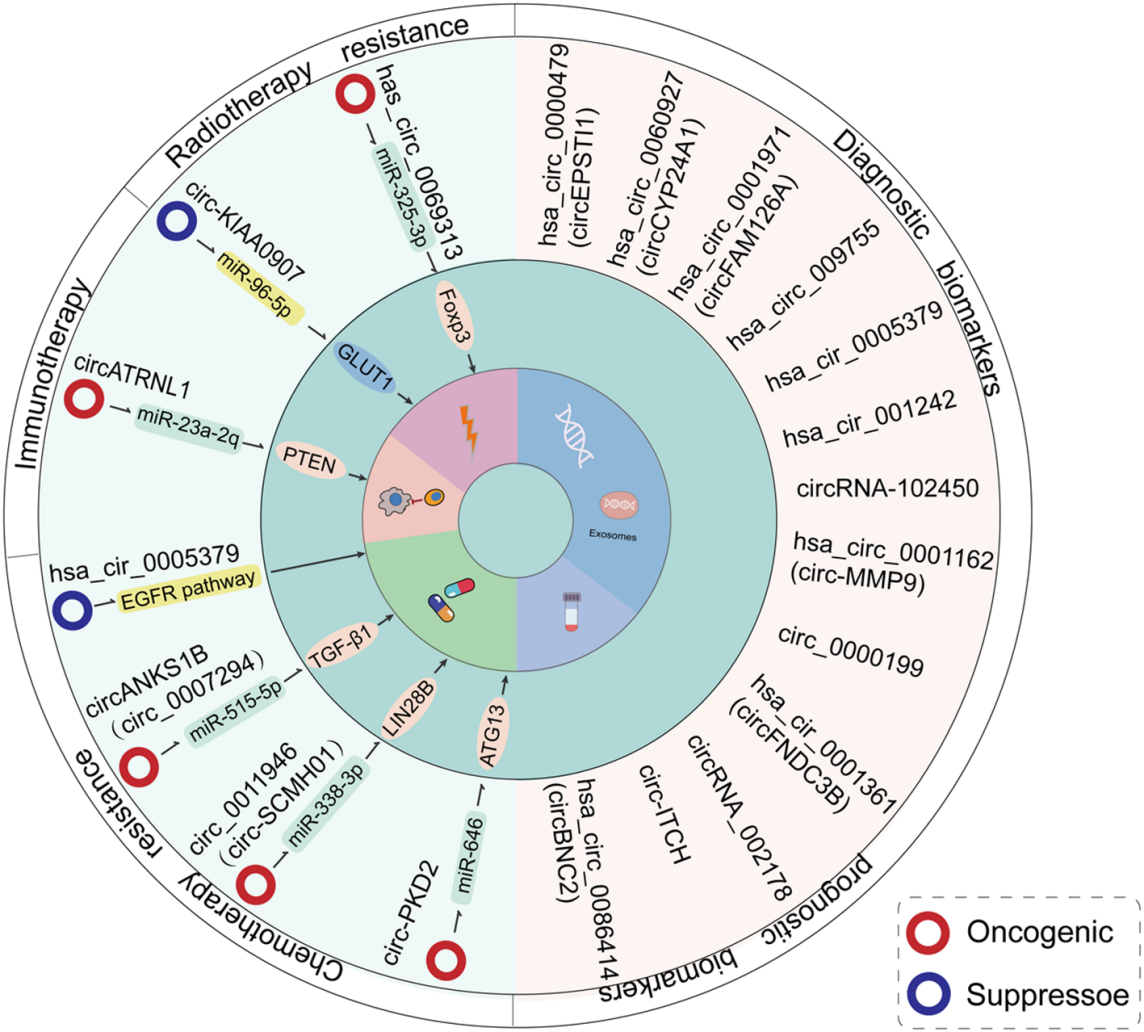
An overview of the clinical functions of circRNAs in the context of OSCC treatment. The expression levels of circRNAs are associated with resistance to radiotherapy/chemotherapy in OSCC patients. Additionally, certain circRNAs may constitute effective diagnostic and prognostic markers for the disease.

Radiation therapy and chemotherapy are key treatment strategies for OSCC; however, the development of drug resistance significantly worsens patient prognosis. Notably, compared to normal tissues, dysregulated circRNAs in OSCC tissues may contribute to the regulation of tumor sensitivity to chemoradiotherapy (**Table 2**, **Figure 8**).

**Table 2:** The characteristic of circRNAs in radiotherapy/chemotherapy resistance of OSCC patients.

circBase_ID	Expression (T vs N)	Host gene	Targets	Anti-tumor effects	Cell lines	Ref.
hsa_circ_0007294	Up	ANKS1B	miR-515-5p/TGF-β1	Cisplatin sensitivity(+)	CAL-27 SCC-9	(67)
hsa_circ_0011946	Up	SCMH1	miR-338-3p/LIN28B	Cisplatin sensitivity(+)	SCC-15 CAL-27	(105)
hsa_circ_0070401	Down	PKD2	miR-646/ATG13	Cisplatin sensitivity(+)	SCC-15 CAL-27	(79)
hsa_circ_0005379	Down	GD12	EGFR	Cetuximab sensitivity(+)	SCC-25 CAL-27	(106)
hsa_circ_0020093	Down	ATRNL1	miR-23a-3p/PTEN	Radiosensitivity(+)	HSC-3 SCC-25	(80)
hsa_circ_0000140	Down	KIAA0907	miR-96-5p/GLUT1	Radiosensitivity(+)	HSC-6 OECM-1	(107)
hsa_circ_0069313	Up	PACRGL	miR-325-3p/Foxp3/PDL1	Immune escape(+)	CAL-27 SCC-9	(70)

Cisplatin (CDDP), a first-generation platinum-based anticancer drug approved by the US FDA in 1978, remains a cornerstone of clinical treatment (108). Despite its broad anticancer efficacy, drug resistance driven by tumor heterogeneity significantly limits its clinical effectiveness (67). Recent studies have highlighted the role of circRNAs, acting as sponges for microRNAs, in mediating CDDP sensitivity in OSCC by regulating various target genes or biological processes. For instance, the oncogenic circANKS1B promotes OSCC metastasis and CDDP resistance by upregulating TGFβ1 *via* sponging miR-515-5p. Knockdown of circANKS1B using siRNA increases OSCC cell sensitivity to CDDP (67). Additionally, circSCMH1 is high expressed in CDDP-resistant OSCC tissues and cell lines. CircSCMH1 can be transferred to surrounding tumor cells *via* exosomes, where it regulates the miR-338-3p/LIN28B axis, driving malignant progression and CDDP resistance in OSCC (105). Another study found that CDDP treatment significantly increases circ-PKD2 expression in OSCC cells. Mechanistically, after overexpression, circPKD2 sponge miR-646 and thus promoting ATG13 expression, triggering autophagy, inducing tumor cell apoptosis, and enhancing chemotherapy sensitivity (79).

Cetuximab is an effective therapy for advanced OSCC. Su et al. demonstrated that circGD12 is involved in Cetuximab resistance, and overexpression of circGD12 enhances cell sensitivity to Cetuximab and induces tumor cell apoptosis through regulation of the EGFR pathway (106).

Furthermore, circRNAs have emerged as potential biological targets to enhance the clinical efficacy of radiation therapy. High-throughput sequencing identified circATRNL1, derived from ATRNL1, as downregulated in OSCC cells after 4Gy ionizing radiation treatment. Upregulation of circATRNL1 induces PTEN expression *via* miR-23a-2p, promoting cell apoptosis, cell cycle arrest, and enhancing OSCC cell sensitivity to radiotherapy (80). Additionally, Dong et al. found that circKIAA0907 may serve as a potential target to improve OSCC radiation resistance. By targeting the circKIAA0907/miR-96-5p/GLUT1 axis, circKIAA0907 enhances OSCC radiation resistance and promotes tumor cell apoptosis (107).

## 7 CircRNAs can be used as a diagnostic marker for OSCC

The highly stable, covalently closed structure, along with the tissue-specific and disease-specific expression patterns of circRNAs, positions them as promising diagnostic biomarkers (15, 109). Several studies have confirmed that aberrantly expressed circRNAs in OSCC may serve as biomarkers for both diagnosis and prognosis (**Table 3**, **Figure 8**).

**Table 3:** The characteristic of CircRNAs in diagnosis prognosis of OSCC patients.

circBase_ID (Name, Expression)	Diagnosis		Prognosis		Ref.
	Clinical outcomes	Clinical subjects	Clinical outcomes	Clinical subjects	
hsa_circ_0006877 (circLDLR, ↓)	RLNM (P=0.022) TNM stage (P=0.044) AUC=0.835	Plasma samples from 16 high and 14 low expressed OSCC patients	Not Applicable		(4)
hsa_circ_0001971 (circFAM126A, ↓)	TNM stage (P=0.019) AUC=0.845	Salivary samples from 93 OSCC patients	Not Applicable		(11)
hsa_circ_0002185 (circUHRF1, ↑)	TNM stage (P=0.008)	Tissue samples from 10 high and 10 low expressed OSCC patients	High circUHRF1 expression is correlated with poor OS (P<0.05)	10 high expressed and 10 low expressed OSCC patients	(40)
hsa_circ_0027451 (circMDM2, ↑)	Not Applicable		High circMDM2 expression is correlated with poor OS (P<0.05)	20 OSCC patients	(41)
hsa_circ_0000519 (circRPPH1, ↑)	DM (P=0.010) T stage (P= 0.045) AUC=0.873	Tissue samples from 25 high and 25 low expressed OSCC patients	High circRPPH1 expression is correlated with poor OS (P<0.05)	25 high expressed and 25 low expressed OSCC patients	(45)
hsa_circ_0001361 (circFNDC3B, ↑)	Not Applicable		High circFNDC3B expression is correlated with poor OS (P<0.05)	11 high expressed and 11 low expressed OSCC patients	(46)
	LNM (P=0.0046), AUC=0.7437	40 N_0_ and 64 N_1–3_ OSCC patient; Plasma samples from 35 OSCC patients and 35 healthy controls	High circFNDC3B expression is correlated with poor RFS (P=0.0279)	104 OSCC patients	(47)
hsa_circ_0000479 (circEPSTI1, ↑)	T stage (P=0.001) TNM stage (P=0.002) AUC=0.869	Tissue samples from 1) 72 high and 82 low expressed; 2) 162 paired of OSCC and OSF tissues	High circEPSTI1 expression are correlated with poor OS and PFS OS (p<0.0001, HR=0.34) PFS (p<0.001, HR=0.35)	72 high and 82 low expressed	(49)
hsa_circ_0000579 (circIGHG, ↑)	Not Applicable		High circIGHG expression are correlated with poor OS (p<0.001) and higher RF (p=0.0012)	64 high and 105 low expressed	(51)
hsa_circ_0577725 (circCLK1, ↑)	LN metastasis (P=0.0343) TNM stage (P=0.0305)	Tissue samples from 30 high and 18 low expressed OSCC patients	Not Applicable		(52)
hsa_circ_0000284 (circHIPK3, ↑)	LN metastasis (P=0.007) TNM stage (P<0.001)	Tissue samples from 40 high and 40 low expressed	High circHIPK3 expression is correlated with poor OS (p=0.015, HR=9.77)	41 high and 118 low expressed	(54)
hsa_circ_0001821 (circPVT1, ↑)	Tumor size (p=0.021) TNM stage (p=0.002) AUC=0.787	Tissue samples from 50 OSCC and paired normal subjects	Not Applicable		(55)
hsa_circ_0001946 (circCDR1as, ↑)	LNM (P=0.017) TNM stage (p=0.006)	Tissue samples from 57 OSCC and paired normal subjects	High circCDR1as expression is correlated with poor OS (p=0.0005)	57 OSCC patients	(59)
hsa_circ_0004390 (circLPAR3, ↑)	AUC=0.8996	Tissue samples from 41 OSCC and paired normal subjects	High circLPAR3 expression is correlated with poor OS (p<0.05)	21 high and 20 low expressed	(62)
hsa_circ_0005615 (circNFAT3, ↑)	LNM (P=0.017) TNM stage (p=0.036)	Tissue samples from 23 high and 23 low expressed OSCC patients	Not Applicable		(63)
hsa_circ_0033144 (circBCL11B, ↑)	DM (P=0.041) Tumor size (P=0.023) TNM stage (p=0.047)	Tissue samples from 25 high and 25 low expressed OSCC patients	High circBCL11B expression is correlated with poor OS (p=0.0342)	25 high and 25 low expressed	(64)
hsa_circ_0001162 (circMMP9, ↑)	LNM (P=0.002) TNM stage (P=0.005) AUC=0.91	Tissue samples from 37 high and 37 low expressed OSCC patients; Plasma samples from 16 healthy and 25 OSCC patients	High circMMP9 expression is correlated with poor OS (p=0.0342)	37 high and 37 low expressed	(65)
hsa_circ_0001682 (circFAM126A, ↑)	LNMs (P<0.01)	Tissue samples from 21 high and 9 low expressed OSCC patients	High circFAM126A expression is correlated with poor OS (p=0.0441)	21 high and 9 low expressed OSCC patients	(44)
hsa_circ_0060927 (circCYP24A1, ↑)	AUC: OSCC vs OLK=0.799 AUC: OSCC vs Nor. = 0.925	Tissue samples from 24 normal, OLK, and OSCC subjects	Not Applicable		(67)
hsa_circ_0001141 (circITCH, ↓)	LNM (P=0.035) TNM stage (P=0.027)	Tissue samples from 46 high and 57 low expressed OSCC patients	Low circITCH expression is correlated with poor OS (p=0.01)	46 high and 57 low expressed OSCC patients	(71)
hsa_circ_0000140 (circKIAA0907, ↓)	LNM (P=0.015) TNM stage (P=0.031)	Tissue samples from 28 high and 28 low expressed OSCC patients	Low circKIAA0907 expression is correlated with poor OS (p<0.001)	28 high and 28 low expressed OSCC patients	(73)
hsa_circ_0006988 (circLDLRAD3, ↓)	LNM (P=0.029) TNM stage (P=0.018)	Tissue samples from 39 high and 30 low expressed OSCC patients	Not Applicable		(76)
hsa_circ_009755 (circVWA8, ↓)	T stage (P=0.011) AUC=0.782	Tissue samples from 27 OSCC tissues and adjacent normal subjects	Not Applicable		(80)
hsa_circ_0093229 (circTRDMT1, ↓)	Tumor size (P=0 0125) T stage (P=0.0317) AUC=0.784	Tissue samples from 40 OSCC tissues and adjacent norma subjects	Not Applicable		(108)
hsa_circ_0000199 (circAKT3, ↑)	Tumor size (p=0.001) LNM (p=0.0295) TNM stage (p=0.0298)	Serum samples from 108 OSCC and 50 healthy subjects	High circAKT3 expression are correlated with poor OS (p<0.0001), DFS (p<0.0001), and higher RF (p=0.0042)	68 high and 40 low expressed OSCC patients	(68)
hsa_circ_0086414 (circBNC2, ↓)	TNM (P=0.047), Tumor size (P=0.012), LNM (P=0.016) AUC=0.749	Tissues from 55 OSCC and adjacent normal subjects	Not Applicable		(81)
hsa_circ_0069313 (circPACRGL, ↑)	Not Applicable		High circPACRGL expression is correlated with poor OS (p<0.0001)	20 high and 30 low expressed OSCC patients	(69)

DM, Distant metastasis; LNM, Lymph node metastasis; OLK, oral leukoplakia; RFS, recurrence-free survival; OS, overall survival; OSF, oral submucous fibrosis; RLNM, regional lymph node metastasis; RF, Recurrence Frequency; DFS, Disease-Free Survival; ↑ means: Compared with normal tissues, circRNA was upregulated in OSCC; ↓ means: Compared with normal tissues, circRNA was downregulated in OSCC.

### 7.1 Screening and diagnosis

Screening and early diagnosis are essential for improving the survival rates and reducing the mortality of patients with OSCC. Oral leukoplakia (OLK) and submucosal fibrosis (OSF) are common precancerous lesions in the oral cavity. **Table 3** summarizes circRNAs that may serve as potential markers for OSCC (**Figure 8**). In general, 18 circRNAs were considered as OSCC candidate biomarkers for clinical screening and diagnosis, including 12 overexpressed and 6 downregulated circRNAs in OSCC tissues compared to adjacent normal or healthy control samples. For instance, high expression of circUHRF1 [40] and circFAM126A (45) in OSCC tissues correlates with the TNM stage (Tumor-Node-Metastasis) (p = 0.008) and LNM (lymph node metastasis, p < 0.01), respectively. The dysregulated expression of these circRNAs in OSCC tissues is associated with TNM stage [circUHRF1 (41)], LNM [circFAM126A (45)], or both [circCLK1 (53), circHIPK3 (55), circCDR1as (60), circNFAT3 (64), circITCH (72), circKIAA0907 (74), and circLDLRAD3 (77)]. In addition to their correlation with tumor stage and metastasis, the tumor expression levels of circPVT1 (56) and circTRDMT1 (80) are associated with tumor size. A study examining the correlation between circRNA expression and early OSCC lesions (including normal buccal mucosa, OSF, and OSCC tissues) found that high expression of circEPSTI1 significantly correlates with T stage (p = 0.001) and advanced TNM stage (p = 0.002). ROC curve analysis (AUC) demonstrated that circEPSTI1 could sensitively differentiate OSCC from OSF (AUC = 0.869) (39). Moreover, circCYP24A1 expression is increased in OSCC, with ROC analysis showing an AUC of 0.799 (95% CI = 0.633-0.916) for OSCC *versus* OLK, and an AUC of 0.925 (95% CI = 0.846-1.0) for OSCC versus normal tissue, suggesting its potential as an early diagnostic biomarker for OSCC (78). Additionally, circBNC2 expression is negatively associated with TNM stage, LNM, and tumor size (all of the p-values were below 0.05), with a potential diagnostic value shown by an ROC AUC of 0.749 (p < 0.0001) (83).

### 7.2 Liquid biopsy

Compared to surgical biopsy, liquid biopsy has gained significant attention in cancer diagnosis and prognosis research due to its real-time, rapid, and minimally invasive nature. Recent studies have explored the correlation between clinical characteristics of OSCC and circRNA expression in body fluids, including saliva, plasma, and serum. CircRNAs in these fluids demonstrate higher sensitivity and specificity compared to tissue samples (110). A circRNA microarray combined with droplet digital PCR (RT ddPCR) identified that circLDLR was a tumor suppressor whom simultaneously related to TNM stage (p = 0.044) and LNM (p = 0.022) in OSCC (4). Similarly, circMMP9 levels were significantly elevated in OSCC plasma samples (the AUC value was 0.91, 95% CI: 0.8216–0.9984) and its expression was associated with TNM stage (p = 0.005) and LNM (p = 0.002) (66). Additionally, circFNDC3B expression was markedly higher in OSCC patients’ serum, with an AUC of 0.7437. High circFNDC3B expression correlated with LNM in 104 patients with OSCC (p = 0.0046) (48). After analyzed the circRNA expressions in serum exosomes from 108 OSCC patients, circAKT3 was screened out due to high expressed circAKT3 in serum was associated with tumor size, TNM stage, and LNM (all of the p-values were below 0.05) (69). Moreover, in clinical salivary samples from 93 patients with OSCC, the levels of circFAM126A were closely related to TNM stage (p = 0.019), and the AUC value was 0.845 (95% CI: 0.784–0.905, p < 0.001) (11).

## 8 CircRNA can be used to analyze the prognosis of OSCC patients

In addition to their diagnostic potential, aberrant circRNA expression in OSCC also serves as a prognostic marker (**Table 3**, **Figure 8**). Previous studies have identified 11 overexpressed and 2 underexpressed circRNAs in OSCC, which correlate with poor overall survival (OS). These include overexpressed circUHRF1 (33), circMDM2 (34), circRPPH1 (36), circFNDC3B (37), circHIPK3 (44), circCDR1as (49), circLPAR3 (52), circBCL11B (54), circMMP9 (56), circFAM126A (57), and circPACRGL (77); and downregulated circITCH (60) and circKIAA0907 (62). In addition to OS, Kaplan-Meier analysis revealed that high expression of circIGHG and circFNDC3B was associated with higher recurrence frequency (RF, p = 0.0012) (41) and poor recurrence-free survival (RFS, p = 0.0279) (58), respectively. Furthermore, compared to low-circAKT3-expressed OSCC patients, high circAKT3 expression in serum exosomes was linked to lower OS and DFS (p-values were below 0.0001) (82).

## 9 Conclusion

The difficulty in early diagnosis, coupled with a high incidence of metastasis and recurrence, results in poor clinical outcomes for patients with OSCC. CircRNAs were initially considered as mere “by-products” of gene transcription. However, accumulating evidence implies that circRNAs act as a crucial factor in regulating the pathological and physiological processes of various diseases, prompting a re-evaluation of these previously overlooked “wastes” (50, 52, 111).

As research on circRNAs in OSCC deepens, multiple studies have highlighted that differentially expressed circRNAs function as either inhibitors or promoters involved in various stages of OSCC tumorigenesis. These processes include sustained cell proliferation, apoptosis resistance, angiogenesis, invasion, metastasis, metabolic reprogramming, immune escape, and acquisition of drug resistance. These findings suggest that circRNAs hold potential as therapeutic targets. Indeed, previous studies have demonstrated that overexpression or knockdown of oncogenic circRNAs has significant anti-tumorigenic effects in OSCC by modulating the expression of target genes (53, 57, 61). Moreover, the stable expression and potential to encode proteins or peptides make circRNAs promising for therapeutic applications. According to recent research, RNA-targeting type VI CRISPR systems (Cas13a, Cas13b, and Cas13d) are capable of directly cleaving single-stranded RNA in mammalian cells. Among them, RfxCas13d has been identified as the most effective system for circRNA interference (112). Artificial circRNAs expressing relevant antigens have shown therapeutic and prophylactic effects in several malignancies (113).

Screening and early diagnosis are critical for improving the survival rate of patients with OSCC (4, 106). However, current diagnostic methods, such as surgical biopsy, are often used to confirm the presence of pathological changes but are unsuitable for early screening. Additionally, established tumor markers such as CEA (carcinoembryonic antigen), ESM-1 (endothelial cell-specific molecule-1), and SCC (squamous cell carcinoma antigen) are not fully applicable for clinical diagnosis of OSCC due to their limited sensitivity and specificity (114, 115). Several studies have shown that dysregulated circRNAs in tumors and body fluids exhibit stage-specific expression in OSCC, closely correlating with clinical features such as tumor size, distant metastasis, and TNM stage. Notably, circRNAs in body fluids, due to their ease of collection, rapid analysis, and minimally invasive nature, are poised to become a primary focus for clinical translation in the future (116). While current circRNA validation remains limited to cellular and animal models due to technological constraints and high costs, continued advancements in molecular biology technology hold promise for substantial progress in the clinical application of circRNAs. Although current RNA sequencing technologies still face challenges in detecting circRNAs in large-scale samples—particularly those with low abundance—the advancements in third-generation sequencing technologies hold promise in overcoming these limitations. These improvements may accelerate the clinical application of circRNAs as reliable biomarkers.
